# *CYP2D6* and *CYP2C19* Genes Associated with Tricontinental and Latin American Ancestry of Peruvians

**DOI:** 10.2174/1872312815666221213151140

**Published:** 2023-06-21

**Authors:** Angel T. Alvarado, María Saravia, Ricardo Losno, Ricardo Pariona, Ana María Muñoz, Roberto O. Ybañez-Julca, Berta Loja, María R. Bendezú, Jorge A. García, Felipe Surco-Laos, Doris Laos-Anchante, Haydee Chávez, Priscilia Aguilar, Mario Pineda

**Affiliations:** 1 International Research Network in Pharmacology and Precision Medicine, Human Medicine School, San Ignacio de Loyola University, USIL, Lima, 15024, Peru;; 2 Latin American Network for the Implementation and Validation of Clinical Pharmacogenomics Guidelines (RELIVAF-CYTED), Madrid, 28001, Spain;; 3 Institute of Food Science and Nutrition, ICAN, San Ignacio de Loyola University, USIL, Lima, 15024, Peru;; 4 Faculty of Pharmacy and Biochemistry, National University of Trujillo, Trujillo, 13001, Peru;; 5 Faculty of Pharmacy and Biochemistry, San Luis Gonzaga National University of Ica, Ica, 11001, Peru;; 6 Human Medicine, Continental University, 15304, Lima, Peru;; 7 Pharmacy and Biochemistry, FCS, Scientific of the South University, UCSUR, Lima, 15067, Peru

**Keywords:** *CYP2D6*, *CYP2C19*, pharmacogenetics, ethnicity, peruvians, alleles

## Abstract

Precision medicine seeks to individualize the dose from the beginning of pharmacological therapy based on the characteristics of each patient, genes involved in the metabolic phenotype, ethnicity or miscegenation, with the purpose to minimize adverse effects and optimize drug efficacy. The objective was to review studies that describe the association of the *CYP2D6* and *CYP2C19* genes with the tricontinental and Latin American ancestry of Peruvians. A bibliographic search was carried out in PubMed/Medline and SciELO, with various descriptors in Spanish and English.

The results of this review confirm that the ethnic origin of Peruvians is tricontinental due to European (mainly Spanish), African and Asian migration, in addition to Latin American migration, being 60.2% mixed, 25.8% Amerindian, 5.9% white, 3.6% African descent, 1.2% Chinese and Japanese descent, and 3.3% unspecified. Studies on CYP2C19*3, CYP2D6*2, *3 and *6 have been reported in Peruvians, and the frequency is similar to that studied in Ecuadorians and Colombians. The CYP2C19*3, CYP2D6*3, and CYP2D6*6 alleles found in Peruvians are common in Europeans, Africans, and Asians; while CYP2D6*4 in Africans and CYP2D6*2 related to Asians. In some studies, the ethnic/gene association has not been demonstrated; while others have shown a significant association, which is why further investigation is warranted. It is concluded that the studies on *CYP2D6* and *CYP2C19* genes associated with the tricontinental and Latin American ancestry of Peruvians are little, and according to what has been investigated, the CYP2C19*3, CYP2D6*2, *3, *4 and *6 alleles have more related to their ancestry.

## 1 INTRODUCTION

Precision medicine seeks to individualize the dose from the start of pharmacological therapy based on the character-istics of each patient, ethnicity, and genotype/phenotype, with the aim of minimizing adverse effects and optimizing drug efficacy [1, 2]. The *CYP2C19* and *CYP2D6* genes are highly polymorphic and express drug-metabolizing enzymes, and have an impact on drug efficacy and safety [3, 4]. Gene CYP2D6 is located on chromosome 22q13.1, and consists of nine exons and eight introns with more than 135 alleles [4-8], including single nucleotide polymorphisms (SNPs) [9, 10].

CYP2D6*1 (wt; rs16947), CYP2D6*2 (G2850T; rs1135840), CYP2D6*33 and CYP2D6*35 [11-14] are the wild type alleles; while CYP2D6*3, *4, *5, and *6 express isoenzymes with no activity, and CYP2D6*10, *17, *29, and *41 encode enzymes with reduced activity [6, 11, 12]. The most common alleles are CYP2D6*3 (2549A>del; rs 35742686) [6, 15, 16], CYP2D6*4 (1846G>A, rs1065852) [12, 17-22], CYP2D6*5 (CYP2D6del) [22, 23], CYP2D6*6 (1707T>del; rs5030655) [12, 20], CYP2D6*10 (rs1065852, 100C<T) [10, 21, 22], and CYP2D6*17 (T107I, R296C, and S486T) [21]. The *CYP2D6* gene encodes monooxygenase proteins that participate in the phase I metabolism of approximately 25% of drugs in clinical use, such as antivirals, opioid analgesics (codeine, tramadol), beta-blockers, antidepressants (amitriptyline, citalopram, clomipramine, desipramine, doxepin, duloxetine, escitalopram, fluoxetine, fluvoxamine, paroxetine and nortriptyline), antipsychotics (aripiprazole, brexpiprazole, clozapine, flupenthixol, fluphenazine, olanzapine), antiarrhythmics (amiodarone, disopyramide, flecainide), antihistamines and β blockers (Atenolol, bisoprolol, carvedilol), cytostatic and statins [10, 22, 24-28]. Gene *CYP2C19* is located on chromosome 10q24; it has more than 35 allelic variants [25, 29, 30]. The most important are CYP2C19*1 (wild type allele), CYP2C19*2 (rs4244285, c.681G>A) [28, 31], CYP2C19*3 (636G >A) [25, 29-32], CYP2C19*4 (A>G at start codon), CYP2C19*5 (1297C>T), CYP2C19*6 (395G>A), CYP2C19*7 (T>A at 5′ donor splice site of intron 5), CYP2C19*8 (358T>C), CYP2C19*16 (1324C>T) [31], and CYP2C19*17 (rs12248560, g.-806C>T and -3042C>T) [28, 31]. The *CYP2C19* enzyme metabolizes nonsteroidal anti-inflammatory drugs (NSAIDs), anticoagulants (clopidogrel), antidepressants (citalopram, escitalopram, sertraline, amitriptyline, clomipramine, doxepin, imipramine, and trimipramine), proton pump inhibitors (PPIs), and other drugs [3, 4, 25, 28, 32]. The combination of alleles of a person determines their genotype (and diplotype), and depending on the allele, a value is assigned to calculate the diplotype activity score; in turn, the diplotype provides a prediction of the metabolic phenotype and its clinical implication [28]. An individual with CYP2D6*1/*1 diplotype is assigned an activity score of 1.0, and is classified as a normal metabolizer (NM); in them, the plasma level of the drug is within the therapeutic index, that is, above the minimum effective plasma concentration (C_mE_) and below the minimum toxic concentration (C_MT_). CYP2D6*1/*9 diplotype with a score of 0.50 is classified as an intermediate metabolizer (IM), with CYP2D6*3/*3 diplotype of score 0 being a poor metabolizer (PM); in individuals with CYP2D6*3/*3 diplotype, plasma levels are observed that exceed the minimum toxic concentration (C_MT_) with risk of presenting adverse effects. Gene duplication has a score of 2.0 and is considered an ultra-rapid metabolizer (UM), with the drug being rapidly metabolized so it does not exceed the minimum effective concentration, which translates into therapeutic failure (tricyclic antidepressants) or generates adverse effects (due to its rapid conversion of codeine into morphine) [1, 4, 8, 33, 34]. Previously, a bibliographic search was carried out on the CYP450 genes in the Peruvian population, and the same one was carried out in the Medline (PubMed) and SciELO databases; these investigations are scarce, so it is worth encouraging their realization. Despite the limited literature on Peru, a review of the *CYP2D6* and *CYP2C19* genes was proposed because they metabolize most of the drugs used in clinical practice (>25%) [10, 22], as both genes are highly polymorphic (135 allelic variants of *CYP2D6* and 35 allelic variants of *CYP2C19*) [4, 25, 29, 30] because they differ within and between populations [4], being the most frequent in European, African, Asian and Latin American populations that have a direct relationship with the Peruvian population [9], and being involved in the variations of the plasmatic levels of the drugs that are metabolized in the different ethnic groups and admixed populations [24]. Peruvians present genetic diversity due to their tricontinental, Latin American ethnic origin and their regional stratification (Coast, Andes and Jungle) [3, 35], which is another justification for reviewing the literature and integrating it into a single manuscript to increase scientific evidence and enabling it to be used in experimental, descriptive and observational studies on pharmacogenes (*CYP2C19/ CYP2D6*) associated with their ethnicity. The objective was to review studies that describe the association of the *CYP2D6* and *CYP2C19* genes with the tricontinental and Latin American ancestry of Peruvians, which allows explaining the variability of plasma drug levels and initiating the implementation of precision medicine in Peru.

## 2 *CYP2D6, CYP2C19* AND ETHNIC ANCESTRY OF PERUVIANS

Genetic ancestry is the information on the biological descent of an individual, including genetic relationships and historical information on the origin and experience of the remote ancestors of the individual. In the case of Peruvians, different levels of genomic ancestry have been reported in 25 regions of Peru; 83% are of American origin and 17% of European origin, and autochthonous genomic inheritance in Peru is around 80%, which corresponds to a very high prevalence of pre-Columbian genes in the current population [13]. Other studies have shown the ethnic origin of Peruvians, which is tricontinental due to European (mainly Spanish), African and Asian (Chinese and Japanese) migration, to which Latin American migration is added [7, 12, 14, 15], being 60.2% admixed (mixed Amerindian and white), 25.8% Amerindian, 5.9% white, 3.6% African descent, 1.2% Chinese and Japanese descent, and 3.3% unspecified [16]. The population of Lima is predominantly admixed (71%) with tricontinental ancestry (European, African and Asian) [2]; for this type of admixture, pharmacogenetic data from other populations are not applicable, so it is vital to have studies on Peruvians [36]. Table **1** [37-40] summarizes the ethnic origin of Peruvians according to the geographical regions admixed (Coast and Andes), Shimaa (Cusco, Andes), Aymara (Andes of Moquegua, Puno and Tacna) and Ashaninka (Jungle).

## 3 STUDIES ON *CYP2D6* AND *CYP2C19* IN PERUVIAN POPULATION

In this review, we have searched for various studies that associate ethnicity/gene (*CYP2C19* and *CYP2D6*) in the Peruvian population according to their ethnic origin. In this sense, 4 theses have been found to obtain the title of a pharmacist in the study carried out by Vera M.; the presence of the *CYP2D6* gene with the allelic variants CYP2D6*1, *3, *4 and *6 was detected in 20 patients with tuberculosis from the city of Arequipa, Peru [40]. Later, Ortiz conducted a study on 20 patients diagnosed with schizophrenia in the city of Ayacucho; in the said study, he used the same technique and with the same reagent limitations as reported by Vera [41]. Valdivia described the CYP2D6*4 allele in the mestizo population of Lima city, Huarochirí province (Lima), Calca province (Cusco) and Puno city (Puno) [42]. While Valdivia reported the genotypic frequency of CYP2C19*2 (*1/*1: n = 70, 87.5%; *1/*2: n = 6, 7.5%, *p* = 0.42; *2/*2: n = 4, 5.0%, *p* = 0.21) and CYP2C19*3 (*1/*1: n = 66, 82.5%; *1/*3: n = 14, 17.5%, *p* = 0.51; *3/*3: n = 0, 0.0%) for patients with epilepsy and for admixed volunteers from the city of Arequipa [43].

In an investigation carried out by Alvarado *et al.*, the frequencies of the allelic variants CYP2D6*3 and CYP2D6*4 were identified in a sample of the Peruvian admixed population stratified by region, 184 subjects from the coast (Lima, n = 134; Tacna, n = 50) and 50 subjects from the Andes (Junín, n = 50) [12]. Continental ancestry and admixture of Old World (Africa, Asia, and Europe), geographic and ethnic diversity of Native American populations contribute to variation in *CYP2D6* [3]. Table **2** describes the studies of *CYP2C19* and *CYP2D6* genes carried out in Peruvians.

## 4 CYP2D6, CYP2C19 AND TRICONTINENTAL ANCESTORS OF PERUVIANS

In the European population, the CYP2C19*2 allele accounts for 93% [44] and 21% CYP2C19*17 [30]; 17% CYP2C19*17 in Africans [30]; in Eastern Asians (China, South Korea and Japan), the prevalence of CYP2C19*2 is 75%, and 25% CYP2C19*3 [44] is specific to Asian individuals and is not found outside of Asian populations [21]. The frequency of the CYP2C19*2 allele is variable in European and Asian populations, and in Hispanics, it is lower (12.6%) [45]. The distribution of *CYP2D6* allelic variants is wide in ethnic groups throughout the world [9]; in the European Caucasian population, CYP2D6*2, *3 *4, *5 and *6, and *41 predominate [8, 28, 34]; in Caucasian Spanish, CYP2D6*1 represents 31% [46], CYP2D6*4 around 25% [7], 2.0-2.38% for CYP2D6*9 [32, 46]; and in Africans, CYP2D6*17 represents 25-40%, especially in sub-Saharan African blacks [21]. In Asians, CYP2D6*10 represents between 47-70% [3, 8, 10, 11, 21, 27, 34], 1.5% CYP2D6*36 [32], while CYP2D5*5 and CYP2D6*14 are exclusive to these populations [8].

**Fig. (1):**
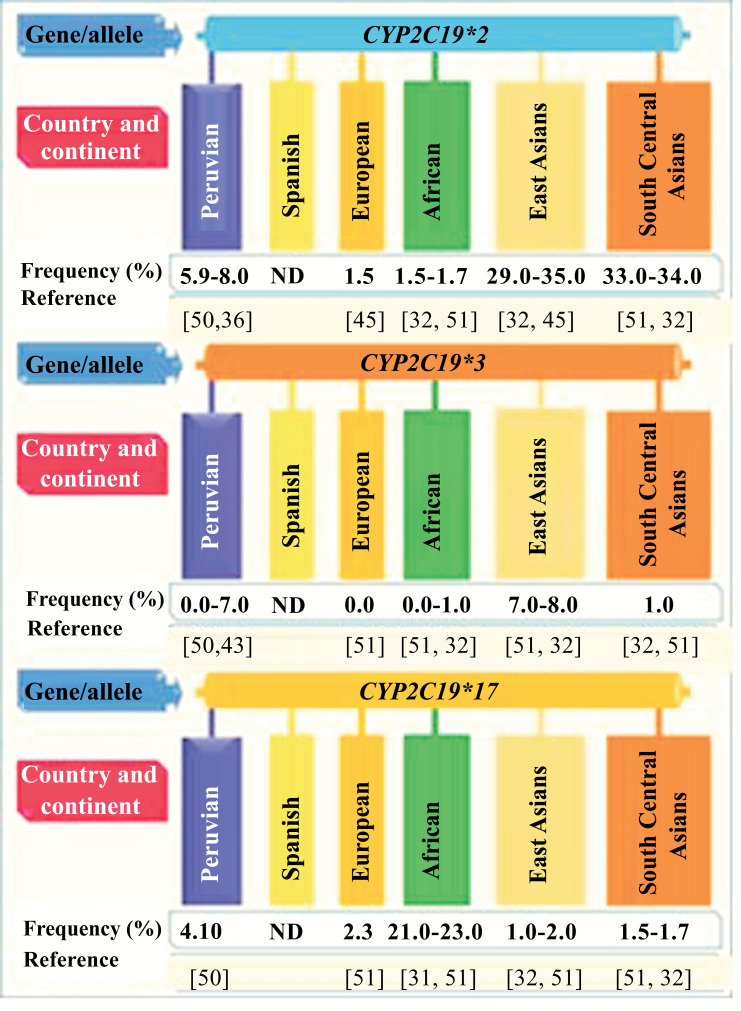
*CYP2C19* allelic frequency of Peruvians and its relationship with their tricontinental ancestry.

The most relevant studies related to the tricontinental ancestry of Peruvians are described below: Wang *et al*. studied *CYP2C19* polymorphism in two provinces (Uygur and Han) of China. A frequency of 5.5% was found for CYP2C19*2/*2 and 30.4% for CYP2C19*1/*2. The metabolic phenotypes are NM in Han 37.7% and in Uygur 40.6%; IM in Han 45.2% and Uygur 34.0%; PM 15.4% in Han and 6.8% in Uygur [47]. Biswas describes that the allele frequencies of CYP2C19*2, *3 and *17 are different in ethnic groups. Africans (37.2%) and Europeans (35.4%) have a higher risk of presenting subtherapeutic effects or adverse effects, which is why they suggest carrying out pharmacogenomic studies of *CYP2C19* and evaluating clinical results [48]. (Fig. **1**) describes the frequency in the percentage of the *CYP2C19* alleles of Peruvians related to tricontinental ancestry.

Menoyo *et al.* studied the CYP2D6 genotype in 105 Spanish-Caucasian volunteers. The percentage is variable for various alleles. CYP2D6*7, *8, *12, *14, *15, and *21 have not been observed [46]. Bousmann *et al.* indicate that CYP2C19*35 alleles are found among African and American ethnic groups (2-9%). They observed the presence of the *CYP2D6* alleles *3, *4, *5, *6, *10, *17, *41. The CYP2D6*29 allele is present in African descendants (6-9%). The frequency of the CYP2D6*12 allele in Peruvians, Colombians, Mexicans, and Puerto Ricans is 2% [32]. Naranjo *et al*. observed wild-type CYP2D6*1 and CYP2C19*1, CYP2D6*41 and CYP2C19*17 (less frequent) alleles in the Native American population [49]. Rodrigues-Soares *et al.* reported that the CYP2D6*2 and CYP2D6*5 alleles are not correlated to continental ancestry. The CYP2D6*35 and CYP2D6*10 alleles are correlated to European ancestry; the *CYP2D6* alleles *17, *29 and *41 have a greater association with continental ancestry. CYP2D6*41 in Ibero-America (0-16.2%) is pre-established in European ancestors [3]. Leitão *et al.* conducted a review of the *CYP2D6* gene in Amerindian populations from Argentina, Costa Rica, Chile, Mexico, Paraguay, Peru, and the United States. The C*YP2D6* allele *17 and *29 were found to be present in Afro-descendant populations [11].

**Fig. (2):**
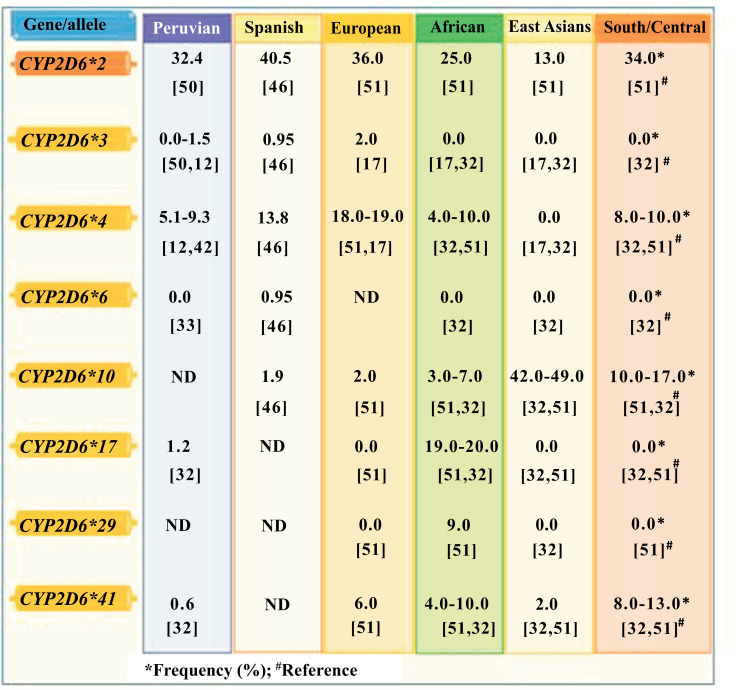
*CYP2D6* allelic frequency of Peruvians and its relationship with their tricontinental ancestry.

Fig. **2** describes the frequency in the percentage of the *CYP2D6* alleles of Peruvians related to tricontinental ancestry.

Regarding the frequency of metabolizers, it has been reported that poor metabolizers (PM) for *CYP2C19* in Caucasian European populations account for 2-5% [27], 4-7% in black Africans [44], 12-23% in Asians [27], specifically 15-17% in Chinese, 18-23% in Japanese, and 12-16% in Koreans [44]. This suggests that the PM phenotype is an autosomal recessive trait that is inherited [44]. Ultra-rapid metabolizers (UM) for CYP2C19*17 are associated with a high risk of bleeding, such as clopidogrel, due to greater exposure to the active metabolite, which inhibits platelet aggregation [30]. PM for *CYP2D6* in Europeans represents between 5-10% [21, 22, 44], 2-5% in African American population, and 0.2%-1.0% in the Asian population [7, 9, 21]. The UM percentage for *CYP2D6* (at least three functional gene copies) in Caucasians is 10%, 3% in African Americans, and 1% in Hispanics, Chinese, and Japanese [34]. Ingelman-Sundberg and Rodríguez-Antona indicate that PMs for *CYP2D6* require 30-50 mg of nortriptyline, and UMs require 500 mg of the drug to achieve the same.

Plasma concentrations. Intermediate metabolizers (IMs) are mainly located in Asia due to the high frequency of CYP2D6*10 [33]. In Amerindian populations from Argentina, Costa Rica, Chile, Mexico, Paraguay, Peru, and the United States, normal metabolizers (NM) for CYP2D6 are the most frequent, followed by IM, PM, and UM in that order [11, 22].

Table **3** summarizes the genes, alleles, diplotype, phenotype, and activity score.

## 5 CYP2D6, CYP2C19 AND LATIN AMERICAN ASCEND-ANCE OF PERUVIANS

Allele frequencies and phenotypes of *CYP2D6* have been extensively studied in all African, European, East Asian, and South Asian populations, with very little in the East African and South Pacific regions [8]. In the Latin American population, it is evident that these studies are still scarce, specifically in Native Americans, despite the importance of pharmacogenetics to individualize pharmacological therapy. Next, a review of the studies carried out in admixed and Amerindian populations is made for the *CYP2C19* and *CYP2D6* genes. Auton *et al.* found in the Bolivian Amerindian population a lower frequency of CYP2C19*2 compared to Caucasians, Asians, Oceanic, and Africans, which could be influenced by their being admixed [50-52]. Santos *et al.* reported the polymorphism of the *CYP2C19* gene according to the ethnic origin of 4 regions of the Brazilian population. CYP2C19*3 and CYP2C19*5 allelic variants were not detected. A frequency of 0.3% of allelic variants of CYP2C19*4 was found, and according to ethnicity, CYP2C19*2 and CYP2C19*17 were observed: Amerindian (10.4%, 15.8%); Caucasians (16.9%, 18.0%); mulattos (16.5%, 21.3%); and Afro-descendants (20.2%, 26.3%), respectively [53]. Salazar-Flores *et al.* observed in five groups of Mexican Amerindians CYP2C19*2 (range 0-31%) and CYP2D6*4 (range 1.2%-7.3%), and CYP2D6*3 was detected exclusively in mestizos. CYP2C19*4 and *5, CYP2D6*6, *7 and *8 were not observed [54]. Favela-Mendoza *et al.* found CYP2C19*2 and CYP2C19*17 (14.29%) in the Mexican admixed population [55]. León-Moreno *et al.* observed the genetic variability of *CYP2C19* in populations from northern and southern Mexico. Frequencies were in the range of 5.9-19.3% for the CYP2C19*2 allele for all populations studied; CYP2C19*3 was not detected [56]. Céspedes-Garro *et al.* observed the presence of CYP2C19*2 and CYP2C9*17 (frequency of 2-10.3%) in mestizo and ancestral Costa Rican populations [24]. Hernandez-Suarez *et al.* reported alleles from the Puerto Rican population with a variable frequency of 14.1% for CYP2C19*17, 13.5% for CYP2C19*2, and 0.3% for CYP2C19*4. When stratifying by geographic region, CYP2C19*2 has been observed with a frequency of 11.7%, 13.6%, and 15% in the central, western, and eastern regions, respectively; CYP2C19*17 was 12.5% in the Central and West and 16% in the East [57]. Flores-Angulo *et al.* observed in the Aragüeño population of Venezuela a frequency of 4.4% of CYP2D6*4, 0.3% of CYP2D6*6, and 1% of CYP2D6*10 [14]. Flores-Gutierrez *et al.* found in the ancestral Warao population a frequency of 0.022% of CYP2C19*2 and 0.0% of CYP2C19*3 [58]. De Andres *et al.* found an overlap in actual enzyme capacity between PM and NM for CYP2D6 (3.14%), and overlap of MU for CYP2C19 (11.48%) and NM for CYP2D6 (2.09%) in Nicaraguan mestizo population [59].

Table **4** describes the relationship of: CYP2C19/CYP2D6 genes of Peruvians with populations of Latin America.

## 6 CLINICAL IMPLICATION OF *CYP2D6* AND *CYP2C19*

Association studies between genotypes/plasma level and adverse drug effects are not conclusive; for example, Carlsson *et al.* reported that CYP2C19*2 and *3 genotypes do not influence plasma levels of citalopram, N-desmethy-lcitalopram, and N,N-didesmethylcitalopram, detected at steady state. The study also concludes that CYP2D6*3, *4, *6 and *2×2 do not influence the plasma levels of citalopram [69]. Thiem *et al.* found a correlation between CYP2C19*1 and the N-demethylation (first nortriptyline, then N-desmethylnotriptyline) and hydroxylation (E-10-hydroxy-N-desmethylnortriptyline) of amitriptyline [70]. While, Aldrich *et al.* observed an association between the metabolic phenotypes and their clinical implication of citalopram and escitalopram [71]. Gasso *et al.* conducted a dose-escalation study of fluoxetine based on metabolic phenotype and found that it influences plasma levels [72]. Bousmann *et al.* indicate that *CYP2C19* poor metabolizers (PM) and *CYP2C19* ultra-rapid metabolizers (UR) require clinical monitoring [32]. Veldic *et al.* performed a retrospective analysis of *CYP2C19* genes in citalopram-treated patients of Caucasian (89.2%), African American (1.1%), Asian (0.7%), and other origins, indicating an association of poor metabolizers with adverse effects [73]. Findling *et al.* noted that clearance of paroxetine is related to *CYP2D6* [74]. While Cherma *et al.* found in two children (of four participants) *CYP2D6* alleles that predict poor metabolizers [75]. Maggo *et al.* carried out a case-control study in patients with depression and inhabitants of European origin (70%), Maori (11%), Asian (4%), and unspecified ethnic groups. PM was observed for *CYP2D6* (n = 15), *CYP2C19* (n = 6), and one individual as PM for *CYP2C19* [76]. Llerena *et al.* have described in Hispanics (excluding Amerindians) PM in a percentage ranging from 0-10%, and UM 0-5.3% [77]. Table **5** summarizes the main studies of genotypes, metabolic phenotype, and their clinical implications.

Fig. (**3**) shows the relationship of plasma concentration according to the metabolic phenotype of the patient.

Plasma concentration *versus* time curves of normal metabolizers (NM: CYP2C19*1 and CYP2D6*1) that are within the therapeutic index; intermediate metabolizers (IM: CYP2C19*1/*2 and CYP2D6*1/*9); ultrarapid metabolizers (UM: CYP2C19*17 and CYP2D6*1xN); and poor metabolizers (PM: CYP2C19*2, *3 and CYP2D6*3, *4), is shown. Figure is made by the authors.

## 7 APPLICATION OF PHARMACOGENOMICS TO PRECISION MEDICINE IN PERU

The results of this review show that studies on *CYP2C19* and *CYP2D6* genes are scarce in Peruvian subpopulations, as pharmacogene association studies, and according to what has been found in the literature, the allelic variants CYP2C19*3, CYP2D6*2 and CYP2D6*3 have a similar frequency in Asians, while CYP2D6*3 and *4 have a greater relationship with Africans. Regarding Latin American descent, there is a greater similarity between Ecuadorians and Colombians. Therefore, it is necessary to carry out studies with a larger number of samples and in the three geographical regions of Peru in order to associate gene allele frequency with the ethnic origin of Peruvians (tricontinental and Latin American) and its influence on the response to drugs; this, at the same time, will allow to have genetic biomarkers typical of Peruvians, and shorten the distance with the other countries of Latin America, where there is more evidence of these studies. In this sense, the Latin American Network for the Implementation and Validation of Pharmacogenomic Clinical Guidelines (RELIVAF), of which Peru is a member, is promoting pharmacogenomic studies in Latin America, and especially in our country, which will allow for greater scientific evidence of the genes, Peruvian genotypes-phenotypes and their association with various drugs, to implement precision medicine in Peru.

The limitations of this review are the few pharmacogenetic studies in patients and the Peruvian population in general, limited studies of the association of ethnicity/genes/drugs, and the heterogeneous design of the international studies that do not allow a significant association between ethnicity and pharmacogenes. Notwithstanding the foregoing, this study will form part of the scientific evidence for Peruvian doctors and researchers to carry out pharmacogenomic studies according to their specialty, and in the short term, it will be a routine clinical practice tool.

## CONCLUSION

It is concluded that the studies on the *CYP2D6* and *CYP2C19* genes associated with the tricontinental and Latin American ancestry of Peruvians are little, and according to what has been investigated, the CYP2C19*3, CYP2D6*2, *3, *4 and *6 alleles have a greater relationship with European, African and Asian populations. Association studies between genotypes/plasma level and adverse drug effects are not conclusive, which is why multicenter studies with a larger number of patients are warranted.

## Figures and Tables

**Fig. (3):**
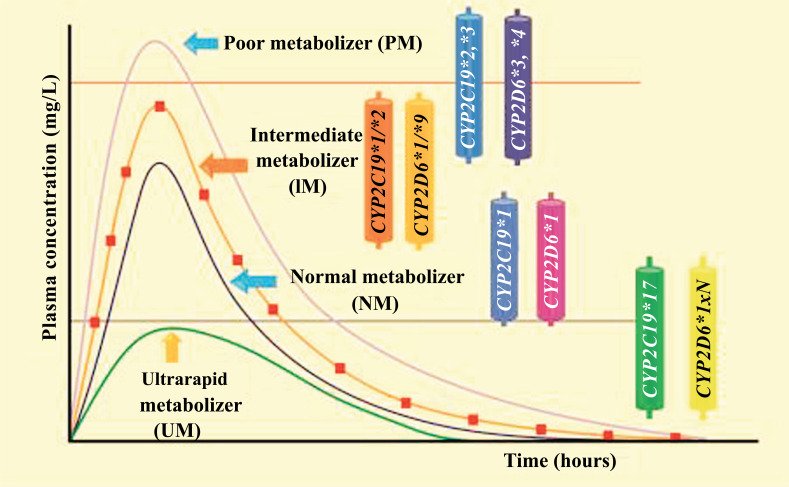
Plasma drug concentration level according to the patient's metabolic phenotype and its clinical implication.

**Table 1:** Ethnic ancestry of Peruvians by European, African, Asian, and Native American migration.

**Peruvian population**	**European (%)**	**African (%)**	**Asian (%)**	**Native American (%)**	**References**
Admixed	24.1-17.0	4.9-3.6	1.2	71.1-83.0	[3, 37, 38, 39]
Shimaa	1.8	0.8	-	97.4	
Aymara	3.2	0.6	-	96.2	
Ashaninka	2.4	0.4	-	97.2	

**Note:** [39] Ethno-linguistic classification of the Peruvian native population; [3] Individuals genotyped for CYP 450 and ancestry.

**Table 2:** Studies of genes associated with ethnic groups and their stratification by regions of Peru.

**Ethnic descent**	***CYP2C19***		***CYP2D6***		**References**
	**Genotype/Nucleotide (n) %**	**Allele (%)**	**Genotype/Nucleotide (n) %**	**Allele (%)**	**-**
Admixed Ayacucho city, Ayacucho	ND	-	*1/*1 A/A (17) 85.0 *1/*4 G/A/G *1/*6 A *1/*3 A/del (3) 15.0	*1 *4 *6 *3	[40]^A^
Admixed Ayacucho city, Ayacucho	ND	-	*1/*1 A/A (18) 90.0 *1/*4 G/G *1/*6 A *1/*3 A/del (2) 10.0	*1 *4 *6 *3	[41]^A^
Admixed Lima city	ND	-	*1/*1 G/G (83) 83.0 *1/*4 G/A (16) 16.0 *4/*4 A/A (1) 1.0	*1 91.0 *4 9.0	[42]
Admixed Huarochiri, Lima	ND	-	*1/*1 G/G (19) 95.0 *1/*4 G/A (1) 5.0 *4/*4 A/A (0) 0.0	*1 97.5 *4 2.5	-
Admixed Calca, Cusco	ND	-	*1/*1G/G (18) 78.3 *1/*4 G/A (5) 21.7 *4/*4 A/A (0) 0.0	*1 89.1 *4 10.9	-
Admixed Puno city, Puno	ND	-	*1/*1 G/G (22) 75.9 *1/*4 G/A (6) 20.7 *4/*4 A/A (1) 3.4	*1 86.2 *4 13.8	-
Admixed Arequipa city, Arequipa	*1/*1 G/G (70) 87.5 *1/*2 G/A (9) 11.3 *2/*2 A/A (1) 1.3 *1/*1 (69) 86.3 *1/*3 (11) 13.8 *3/*3 (0) 0.00	*1 93.0 *2 7.0 *1 93.0 *3 7.0	ND	-	[43]
Admixed Lima city	ND	-	*1/*1 A/A (128) 95.5 *1/*3 A/del (6) 4.5 *3/*3 del/del (0) 0.0 *1/*1 G/G (121) 90.3 *1/*4 G/A (13) 9.7 *4/*4 A/A (0) 0.0	*1 97.8 *3 2.2 *1 95.2 *4 4.8	[12]
Admixed Tacna city, Tacna	ND	-	*1/*1 A/A (50) 100.0 *1/*3 A/del (0) 0.0 *3/*3 del/del (0) 0.0 *1/*1 G/G (45) 90.0 *1/*4 G/A (5) 10.0 *4/*4 A/A (0) 0.0	*1 100.0 *3 0.0 *1 95.0 *4 5.0	-
Admixed Huancayo city, Junin	ND	-	*1/*1 A/A (49) 98.0 *1/*3 A/del (1) 2.0 *3/*3 del/del (0) 0.0 *1/*1 G/G (44) 88.0 *1/*4 G/A (4) 8.0 *4/*4 A/A (2) 4.0	*1 99.0 *3 1.0 *1 94.0 *4 6.0	-

**Note:** ^A^It was not discriminated if they were CYP2D6*1, *4 or *6; ND: Undetermined.

**Table 3:** Alleles, phenotypes, and activity score value of *CYP2C19* and *CYP2D6* diplotypes.

**Gene/ Chromosome: *CYP2C19*/10q24**				**References**
**Allele**	**Diplotype**	**Phenotype**	**Activity**	[24, 25, 29]
CYP2C19*1	CYP2C19*1/*1	NM	1	
CYP2C19*9 CYP2C19*10 CYP2C19*12 CYP2C19*16 CYP2C19*25 CYP2C19*27	CYP2C19*1/*2	IM	0.5	
CYP2C19*2 CYP2C19*3 CYP2C19*4 CYP2C19*5 CYP2C19*6 CYP2C19*7 CYP2C19*8	CYP2C19*2/*2 CYP2C19*2/*4 CYP2C19*4/*4	PM	0	
CYP2C19*17	CYP2C19*1/*17 CYP2C19*17/*17	UM	2	
**Gene/ Chromosome: *CYP2D6*/22q13.1**				**References**
CYP2D6*1 CYP2D6*2 CYP2D6*35	CYP2D6*1/*1 CYP2D6*1/*2 CYP2D6*1/*4 CYP2D6*1/*5 CYP2D6*1/*10 CYP2D6*1/*35 CYP2D6*2/*2	NM	1	[4, 6, 11]
CYP2D6*9 CYP2D6*10 CYP2D6*17 CYP2D6*29 CYP2D6*41	CYP2D6*1/*9 CYP2D6*/*17 CYP2D6*10/*10 CYP2D6*6/*29 CYP2D6*1/*41	IM	0.5	
CYP2D6*3 CYP2D6*4 CYP2D6*5 CYP2D6*6 CYP2D6*40	CYP2D6*3/*3 CYP2D6*3/*4 CYP2D6*4/*4 CYP2D6*4/*5 CYP2D6*6/*6	PM	0	
CYP2D6*1x2 CYP2D6*2x2	*-*	UM	2	

**Table 4:** *CYP2C19/CYP2D6* genes and alleles of Peruvians compared to Latin American populations.

**Gene/** **Allele**	**Peru**	**Bolivia**	**Brazil**	**Colombia**	**Chile**	**Ecuador**	**Argentina**	**Venezuela**	**Mexico**
*CYP2C19* *2	5.9 [50]	7.8 [52]	16.0 [53]	8.7 [60]	12.0 [63]	7.8 [65]	ND	0.14 [58] 0.07 [58]	8.6 [55]
*3	0.0 [50]	0.1 [52]	0.0 [53]	0.0 [60]	0.0 [63]	0.4 [65]	ND	0.013 [58] 0.044 [58]	0.0 [55]
*4	0.0 [50]	ND	0.3 [53]	0.0 [60]	ND	ND	ND	-	0.0 [55]
*17	4.1 [50]	ND	20.4 [53]	12.8 [60]	ND	24.9 [65]	ND	-	14.3 [55]
*CYP2D6* *2	32.4 [50]	ND	ND	3.7 [60]	40.7 [64]	31.4 [66]	17.4 [67]	-	17.8 [68]
*3	0.0 [50]	ND	ND	1.2 [61]	1.1 [64]	0.4 [66]	0.6 [67]	-	1.4 [68]
*4	6.5 [50]	ND	ND	19.4 [61]	11.8 [64]	10.6 [66]	16.4 [68]	4.4 [14]	11.2 [68]
*5	ND	ND	ND	0.8 [61]	ND	2.1 [66]	2.8 [68]	-	0.03 [68]
*6	0.0 [50]	ND	ND	0.1 [62]	ND	0.0 [66]	0.4 [68]	0.3 [14]	ND
*17	1.2 [50]	ND	ND	1.6 [61]	0.0 [64]	0.4 [66]	0.2 [68]	-	1.7 [68]
*41	0.6 [50]	ND	ND	8.0 [62]	ND	2.5 [66]	7.7 [68]	-	2.2 [68]
*1xN or *2xN	ND [50]	ND	ND	ND	0.3 [64]	0.8 [66]	3.4 [68]	-	5.0 [68]

**Note:** *CYP2C19/CYP2D6* genes of Peruvians with populations of Latin America.

**Table 5:** Genotypes, metabolic phenotypes and clinical implications.

**Type of Study**	**Genotype/Metabolic Phenotype**	**Clinical Implications**	**References**
Descriptive study	CYP2C19*2 (G>A, Gly681Ala) CYP2C19*3 (Gly636Ala)	Oral contraceptives may influence the metabolism of citalopram. This could be due to a contraceptive interaction with CYP2C19.	[69]
Descriptive study	*PM para CYP2C19*	In PM for CYP2C19, a significantly lower amount of demethylation was evidenced.	[70]
Retrospective study	Poor metabolizer (PM) CYP2C19*2/*2 CYP2C19*2/*4 Ultra-rapid metabolizers (UR) CYP2C19*1/*17 CYP2C19*17/*17	PM patients present side effects, with citalopram and escitalopram being the most likely cause of discontinuing treatment. UR patients have fewer adverse effects and respond to treatment with citalopram and escitalopram.	[71]
Review study including gene-drug association studies	PM: CYP2C19*2/*2 CYP2C19*2/*4 UR: CYP2C19*1/*17 CYP2C19*17/*17	Individuals with CYP2C19*2/*2 and CYP2C19*2/*4 alleles require lower doses (50% of the recommended dose), and patients with CYP2C19*1/*17 or *17/*17 alleles require an antidepressant that is not metabolized by *CYP2C19.*	[32]
Retrospective study of depressed patients (2003-2013)	PM: CYP2C19*2/*2 CYP2C19*2/*4	Significance in PM was observed for *CYP2C19*. Plasma levels of citalopram/escitalopram were increased with the risk of adverse effects.	[73]
Descriptive study	RM y UR para *CYP2D6*	These genotypes do not influence the plasma levels of citalopram and its two metabolites.	[69]
Descriptive study	*CYP2D6*	*CYP2D6* influences the clearance of paroxetine.	[74]
Open design study	PM: CYP2D6*4/*4	It was observed that it influences the plasmatic level; subsequently, fluvoxamine doses were personalized, being three times lower (75-100 mg) to achieve levels of 58 ng/mL. As it was a very small sample, this association was not statistically significant.	[75]
Descriptive study	*CYP2D6*	The fluoxetine/(S)-norfluoxetine ratio was found to be low in UR, and higher in IM and PM.	[72]
Cases and controls study	PM para CYP2D6 PM para CYP2C19	On 7/15 PM *CYP2D6* were dose intolerants of escitalopram, citalopram, or sertraline. PM *CYP2C19* tolerated escitalopram dose but not fluoxetine (minimally metabolized by CYP2C19).	[76]

## LIST OF ABBREVIATIONS

NM: Normal Metabolizers
NSAIDs: Nonsteroidal Anti-inflammatory Drugs
PM: Poor Metabolizers
PPIs: Proton Pump Inhibitors
UM: Ultra-rapid Metabolizer
UR: Ultra-rapid Metabolizers
